# Enhancing naked oat (*Avena nuda* L.) productivity with minimal indirect nitrogen loss and maximum nitrogen use efficiency through integrated use of different nitrogen sources

**DOI:** 10.1371/journal.pone.0213808

**Published:** 2019-03-18

**Authors:** Tariful Alam Khan, Faisal Nadeem, Lili Chen, Xiaofen Wang, Zhaohai Zeng, Yuegao Hu

**Affiliations:** 1 College of Agronomy, China Agricultural University, Beijing, China; 2 College of Resources and Environmental Sciences, China Agricultural University, Beijing, China; University of Delhi, INDIA

## Abstract

Oat (Avena nuda L.) is a nutritious grain crop, rich in dietary fibers and phytochemicals. Application of efficient nitrogen (N) sources and dose is very important to obtain higher crop productivity and to achieve environmental sustainability. The exploitation of natural beneficial microbes and organic nitrogen in combination with chemical nitrogen would be effective to boost soil N for plant uptake. Hence, a field experiment was conducted during 2016 and 2017 with the aim to ameliorate the use of chemical N (CN) with organic nitrogen (ON) and microbial fertilizer (MBF) without compromising the productivity of oat. T1 = control, T2 = 100% CN, T3 = 100% CN+MBF, T4 = 75% CN+ 25% ON+MBF, T5 = 50% CN+ 50% ON+MBF, T6 = 100% ON+MBF, T7 = 100% ON were the treatments. 50% CN + 50% ON + MBF treatment proved to be an efficient combination regarding enhanced biomass and grain yield, nitrogen uptake and NUE as compared to rest of the treatments in both years. During the critical stages of the crop, when most of the applied CN was leached from the top 20 cm soil depth, a substantial N came from the PM mineralization through enhanced microbial activity by the addition of MBF. Lastly, the application of ON supplemented with MBF improved the rhizosphere soil properties, i.e. mineral N concentration, total N (TN), soil organic carbon (SOC), microbial biomass carbon (MBC), microbial biomass nitrogen (MBN), soil respiration rate and enzymatic activity. A balanced and source conscious application of CN, ON and MBF reduced N losses and added a substantial amount of N into the soil N pool. We concluded that organic N combined with chemical N and MBF proved to be effective in improving soil properties ensuring less N loss and increasing oat production in the semi-arid region.

## Introduction

Agriculture, which plays a pivotal role in meeting the food demands of the growing world population, is becoming increasingly dependent on chemical fertilizers [1], as a good correlation has been found by the application of industrially manufactured fertilizers with crop yields [2,3]. Plant growth and development necessarily depend on nitrogen as it is a crucial component of chlorophyll required for photosynthesis, nucleic acids, amino acids, proteins and some organic acids [4]. However, the over application of synthetic nitrogenous fertilizers is not effective for increasing crop yields, because of higher vulnerability to nitrogen losses in the form of volatilization (emitting oxides of nitrogen), denitrification, leaching, (polluting underground water reserves), and eutrophication (polluting surface water bodies), not only increase the cost of production but also lead to serious environmental health hazards [5,6,7,8]. Hence, high levels of soil N are susceptible to losses [9,10] which ultimately can reduce the N use efficiency (NUE) [11]. Nitrogen losses from NH_3_ volatilization, NO_3_ leaching and denitrification were estimated to be 23, 18 and 2% respectively in wheat-maize rotation system in the North China Plain (NCP). On the other hand, about 50% of the applied CN is lost by different processes in paddy fields [12,13], and the N loss through leaching is the major contributor [14]. Therefore, improved management of the N application rate with appropriate N sources and their combinations is the key to increase crop NUE [15,16,17]. Organic nitrogen (ON) and microbial fertilizers (MBF) in a combination with chemical nitrogen may prove to reduce the cost of production, environmental hazards without compromising crop yield.

Among organic manures, poultry manure (PM) is easily available, environment friendly, rich in nutrients with quick release, capable of increasing soil organic carbon and supplying all essential macronutrients (N, P, K, Ca and Mg) for plant growth and development [18,19]. Although organic manure has the limitations of slow release of nutrients with lesser nutrient utilization, it also has the benefits of boosting soil physiochemical processes by increasing microbial activity.

Microbial fertilizers (MBF) are comprised of living cells of distinct micro-organisms which are usually applied as seed inoculants or liquid spray to the root rhizosphere. These micro-organisms colonize in the root zone and nourish plant growth by converting unavailable fundamental soil nutrients (N, P) into available forms through biological processes [20]. They increase soil microbial biomass (N and C) in green house [21] as well as field conditions [22]. The microbes especially bacteria, present in microbial fertilizer, not only benefit leguminous crops through nodulation and atmospheric nitrogen fixation, but their beneficial association with non-legumes especially cereals has also been reported [23]. However, it is not possible to rely on MBF only by completely replacing the use of chemical fertilizer, due to their unequal distribution in soil [24,25]. The intermingling of microbial fertilizers and chemical fertilizers in different agro climatic conditions of different countries revealed a higher performance of microbial fertilizers in the presence of lower dose of chemical fertilizers [26].

There have been previous studies reporting nitrogen use efficiency in cereals like maize, rice, wheat and foxtail millet [27,28,29,30]. Naked oat (*Avena nuda* L.) and hulled oat (*A*. *sativa* L.) are two types of oats belonging to the *Avena* genus and Gramineae family cultivated in China. The fast-growing nature of oats (*Avena nuda* L.) gives them the capacity to produce ample quantities of fresh fodder within a short period with high nutritional values. The high protein and β-glucone content along with the presence of hypo-glycemic fiber content in oat grain increase its value as a human food. On the other hand, rice, wheat, maize and other high yielding cereal crops generally occupy most of the agricultural land but oat can be cultivated in marginal land with less fertilizer and water requirements.

This study aims to reduce the use of chemical N fertilizers, obtain high N use efficiency and limit environmental impact from polluting. The main objectives of this experiment were to evaluate yield of naked oat under the provision of chemical N and organic N supplemented with microbial fertilizer, to examine the NUE in oat production system, and to explore the changes in soil N, C and enzymatic activity.

## Materials and methods

### Experimental site description

The experiment was conducted during 2016 and 2017 at Baicheng Academy of Agricultural Sciences in Baicheng City (45° 37’N, 122° 48’E, 155 m above sea level), Jilin province, China. No human, animal or plant species were harmed during the experiment. The region is a typical semi-arid area located in Northeast China, with a mean annual precipitation of 407 mm (mostly occurs from April to September). The area has a temperate, semiarid and continental climate, with a mean temperature of 18.02°C during the cropping season and a frost-free period of 125–135 days. The average annual effective accumulated temperature is 2915°C. The soil is light Chernozem. The former crop was sunflowers. The basic soil physicochemical properties and average daily air temperature and daily rainfall during the oat growing season at the study site (April to October) are shown in **S1 Fig**.

### Experimental treatments, design and field management

The experiment comprised of seven fertilizer treatments: T1 = control- without chemical nitrogen (CN), organic nitrogen (ON) and microbial fertilizer (MBF), T2 = 100% CN, T3 = 100% CN + MBF, T4 = 75% CN + 25% ON + MBF, T5 = 50% CN + 50% ON+ MBF, T6 = 100% ON + MBF, T7 = 100% ON. The experimental arrangement was a randomized complete block design (RCBD) with four replications. There were 28 plots in total. Every plot had a size of 20 m^2^ (5 m × 4 m) and 12 rows with row to row distance of 30 cm. Every plot received 300 g of oat seeds (25g or around 750 seeds/row). The recommended dose of N (i.e. 90 kg N ha^−1^ as practiced by the farmers in this area) was considered as 100% dose. High quality seeds of oat cultivar “Bai Yan 2” were obtained from Baicheng Academy of Agricultural Science, at Jilin Province in China.

### Fertilizer management

#### Chemical N and other nutrients application

Urea (46.3% N) was used as a source of chemical N, and 90, 67.5 and 45 kg N ha^-1^ were applied as 100, 75 and 50% N doses. In addition, P was applied at the rate of 55 Kg ha^-1^ P_2_O_5_ Whereas K was applied at the rate of 45 Kg ha^-1^ K_2_O. The source of P was Ca (H_2_PO4)_2_·H2O and that of K was K_2_SO_4_, which were also applied to all experimental treatments including control (**S3 Table**). N, P, and K were applied as basal dose, broadcasted and incorporated into the top 20 cm soil layer before seed sowing.

#### Organic fertilizer application

A composted, crushed, sieved (<1mm) and properly mixed poultry manure (PM) was collected from Shijiazhuang animal breeding station, Hebei province, China and used as a source of ON. The N, P and K content of the poultry manure were 18.68 g kg^-1^, 8.17g kg^-1^ and 5.41 g kg^-1^, respectively.

#### Microbial fertilizer

Microbial fertilizer with the effective viable count of ≥ 2 million g^-1^ used in this study was supplied by the Beijing Liuhe Shenzhou Biotechnology Co., Ltd. It was a mixed microbial fertilizer with lignite as the base material. Microbial fertilizer was applied at the rate of 20 kg ha^-1^ and was divided into two slots; First, the seed was inoculated with 15 kg ha^-1^ MBF and secondly, it was sprayed (5 kg ha^-1^ MBF mixed with water) in the rhizosphere just after tillering stage. The high-throughput sequencing of the MBF was done by Shanghai Meiji Biomedical Technology Company. High throughput sequencing was performed using the MiSeq sequencing platform. DNA was extracted from 0.3 g of fresh soil using the E.Z. N.A. Soil DNA Kit Omega Bio-teck Inc. Norcross, GA, USA, according to manufacturer’s instructions. DNA from each sample was extracted in three replicates and pooled to form one mixed DNA sample. The extracted DNA was checked on a 1% agarose gel and the concentration was determined using a NANO Quant (Tecan, Männedorf, Switzerland). Bacterial consortia were further analyzed by sequencing the V3-V4 hypervariable region of the 16S rRNA gene. Universal primers 338F (5´-ACTCCTACGGGAGGCAGCA-3´) and 806R (5´-GGACTACHVGGGTWTCTAAT-3´) were used to amplify V3-V4 region. Whereas, 817F (5’-TTAGCATGGAATAATRRAATAGGA-3’) and 1196R (5’-TCTGGACCTGGTGAGTTTCC-3’) were the primers used to amplify the hypervariable regions of the fungal 18S rRNA gene using a thermocycler PCR system (GeneAmp 9700, ABI, U.S.A.). A reaction mixture, containing 5X FastPfu Buffer, dNTPs (2.5 mM), forward and reverse primers (5 μM), FastPfu Polymerase and template DNA was prepared in triplicate for PCR reaction. The PCR reactions were conducted using the following program: denaturation at 95°C for 3 min followed by 35 cycles of 30 s at 95°C, annealing at 55°C for 30 s, elongation at 72°C for 45 s, with a final extension of 10 min at 72°C. The results of the high-throughput sequencing of the beneficial functional flora contained in the microbial fertilizers used in this experiment are displayed in **S2 Fig.** For detecting the relative abundance of *nif*H gene the PCR amplification of *nif*H gene was performed on an ABI GeneAmp 9700 PCR thermocycler (Applied Biosystems) using the primers *nif*H-F/*nif*H-R (5′-barcode-AAA GGY GGW ATC GGY AARTCC ACC AC-3′/5′-TTG TTS GCS GCRTAC ATS GCC ATC AT-3′[31]. Seeds were soaked in water overnight dried on blotting paper and mixed with microbial fertilizers and finally sown in the field manually.

### Sampling, measurements and chemical analysis

#### Plant sampling and analysis

Plant samples were collected at harvesting stage to define the aboveground biomass (including grain weight). From each experimental plot, above ground plant parts were harvested randomly from an area of 100 cm^2^ in duplicate (as two technical replicates) with four replicates per treatment to estimate aboveground fresh biomass yield. After that, each sample was weighed and then oven-dried at 105°C for 30 min and then dried at 70°C for 3 days to get constant weight to calculate the moisture gravimetrically and to estimate dry biomass. For grain yield, an area of 2 m^2^ (edge rows were avoided) was selected randomly from each plot (in duplicate), and all plants were harvested, sun dried, and threshed to get grains. A moisture meter was used to measure the grain moisture contents, and grain yield was standardized at 12.5% moisture content. All the yield contributing parameter like plant height, ear length, effective spike number per hectare, grain number per spike, and 1000 grain weight were also investigated at maturity. After that, grains were oven-dried at 105°C for 30 minutes and then at 75°C until constant weight. Oven dried Straw and grains were ground into powder using a Wiley mill, passed through a 0.5-mm mesh and stored at 4°C for N analysis. The total N content in straw and grains was estimated by a semi-micro Kjeldahl digestion and distillation method [32]. The concentration of other mineral elements (P, K, Ca, Mg, Mn, Zn, Cu and Fe) in root and shoot were determined using ICP-MS according to the method described by Masson et al., (2010) [33].

#### Soil sampling and analysis

Concurrently, soil samples (0–20 and 20–40 cm soil depth) were also collected at pre-seeding, tillering, jointing-booting and after final harvest stage. The samples were taken from 10 different points randomly from each plot and homogenized to get a composite sample. These composite soil samples were sieved through 2 mm mesh, and moisture content was determined. Soil moisture content was determined gravimetrically after drying for 24 hours at 105°C. Each soil sample was divided into two parts. One part was placed in plastic bag and stored in a refrigerator at -4°C to analyze mineral N, dissolved organic carbon (DOC), dissolved organic nitrogen (DON), microbial biomass Nitrogen (MBN), microbial biomass carbon (MBC) and enzymatic activity. The other part of the soil sample was air dried, preserved in plastic bags and placed in a refrigerator at 4°C for the analysis of total N, organic carbon, and pH. Soil pH was measured by preparing 1:2.5 soils: water suspension. Soil NH_4_^+^-N and NO_3_^-^-N were analyzed by using a continuous flow analyzer (AA3 type, company: Seal, Norderstedt, Germany). Walkley-Black method was used to determined soil organic carbon (SOC) [34]. Soil microbial biomass C and N were detected by chloroform fumigation- extraction method [35]. After destructive sampling, 20 g of carefully mixed soil was directly extracted using 80 ml of 0.5 M K_2_SO_4_ by shaking for ½ h at 180 revs/min. Another 20 g soil was fumigated with chloroform for 24 h and extracted in the same manner. The extracts were analyzed for the total C concentration using a 2100 TOC/TIC analyzer (Analytik Jena, Germany). The extracts of the non-fumigated samples were used to detect DOC and DON [36]. The biomass was calculated based on the difference of K_2_SO_4_ extract between fumigated and non-fumigated soil samples by applying two conversion factor 0.54 for MBN (*k*_*EN*_) and 0.45for MBC (*k*_*EC*_), in the following equation [37].

$$\begin{array}{l} MBC=\frac{E_{C}}{k_{EC}}\\ MBN=\frac{E_{N}}{k_{EN}} \end{array}$$

Where, *E*_*C*_ and *E*_*N*_ refer to the different organic carbon and total N amount between fumigated and non-fumigated treatment, respectively.

The soil respiration (SR) was estimated by the sealed jar incubation method, where the soil was pre-incubated at 20°C for 7 days in dark condition by adjusting 55% water holding capacity and measured the rate of CO_2_ derived from the moist soil. NaOH trap was used to measure the CO_2_ for next 7 days and then titrated with HCl [38].

#### Enzymatic activities of soil

The activity of dehydrogenase (DHA, Enzyme Commission number 1.1.1) enzyme was measured by using 2, 3, 5-triphenyltetrazolium chloride as a substrate [39]. α-benzoyl-N-argininamide was used as a substrate to detect hydrolyzing benzoyl argininamide (BAA-protease EC 3.4.4) [40]. *p*-nitrophenyl phosphate was used to determine acid phosphatase (EC3.1.3.2) [41], while *p*-nitrophenyl-β-d-glucopyranoside and *p*-nitrophenyl sulphate were used as a substrate to measure β-glucosidase [42] and arylsulphatase [43], respectively.

#### Calculation

Nitrogen uptake in straw and grain was calculated by multiplying the nitrogen concentration with its dry weight. The components of NUE were calculated with the following formulas:

Partial factor productivity (marginal) (PFP; kg kg^-1^ N) = $\frac{Y}{NA}$

Agronomic nitrogen use efficiency (marginal) (ANUE; kg kg^-1^ N) = $\frac{Y-Y0}{NA}$

Apparent nitrogen recovery efficiency (marginal) (ANRE; %) = $\left\{\frac{U-U0}{NA}\right\}\times 100$

Physiological efficiency (marginal) (PNUE; kg kg^-1^ N) = $\frac{Y-Y0}{U-U0}$

Y = yield of a harvested portion (grain+ biomass) of the crop with N applied; Y0 = yield without N application; NA = amount of total N applied (CN + ON); U = total N uptake by crop with N application; U0 = total N uptake by crop without N application

### Statistical analysis

Statistical analysis was performed using a windows software package (IBM SPSS Statistics 22). Means of different treatments were compared using the least significant difference at a 0.05 or 0.01 level of probability. Correlation analysis was performed using Pearson correlation function of SPSS. Graphical rendering was carried out using Sigma plot 13.0 windows software. All the data are presented as means ± standard errors (SE) of four replicates.

## Results

### Two-Way ANOVA analysis

**Fig 1:**
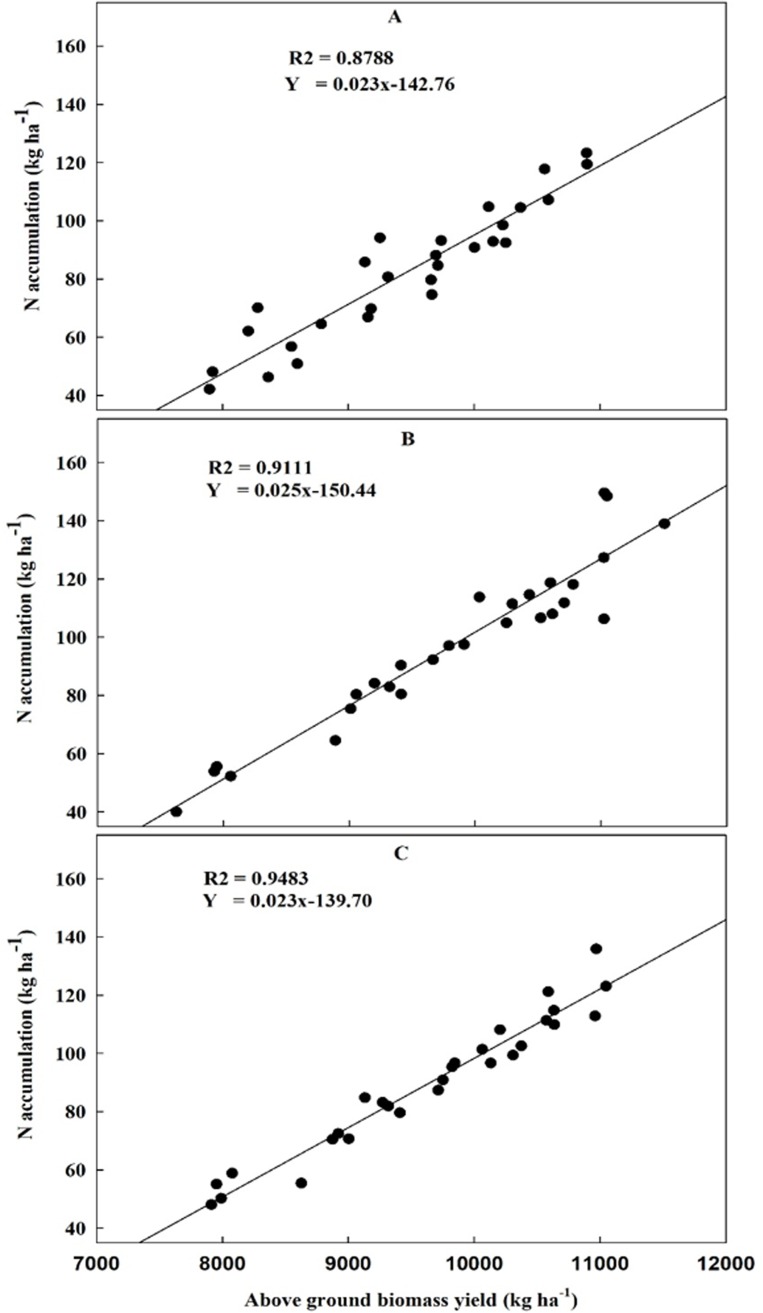
Above ground biomass with respect to N accumulation. The relation between above ground biomass yield and N accumulation in the year (A) 2016, (B) 2017 and (C) both year pooled data.

Analysis of variance showed the significance of treatments on all parameters except N concentration in grain and physiological nitrogen use efficiency (PNUE). Years had mixed trend with above ground biomass yield, grain weight straw weight, N concentration in straw, N accumulation in above ground plant parts and N accumulation in straw showing significance while the rest were non-significant. The interactive effect of year and treatment was significant for partial factor productivity (PFP), agronomic nitrogen use efficiency (ANUE), apparent N recovery efficiency (ANRE) and above ground biomass yield while non-significant for others (**Table 1**). A strongly positive correlation between N accumulation and above ground biomass yield was found in the year 2016, 2017 and both (**Fig 1A, 1B and 1C**).

**Table 1:** The analysis of variance (ANOVA) for the year and treatment effects and their interaction on the above ground weight, above ground nitrogen concentration, accumulation and nitrogen use efficiency of oat.

Parameter	Year	Treatment	Year * Treatment
Above ground biomass yield	24.370***	155.941***	3.223**
Grain weight	9.786**	101.4***	1.070ns
Straw weight	11.178**	49.043***	1.718ns
N concentration in aboveground plant parts	2.81ns	3.75**	0.190ns
N concentration in grain	1.239ns	1.785ns	0.367ns
N concentration in Straw	10.616**	11.395***	0.707ns
N accumulation in aboveground plant parts	15.293***	29.486***	1.463ns
N accumulation in grain	3.190ns	10.113***	0.479ns
N accumulation in Straw	8.983**	11.700***	0.636ns
Partial Factor Productivity (PFP)	0.000ns	31.196***	64.748***
Agronomic N use efficiency (ANUE)	2.743ns	21.056***	39.608***
Apparent N recovery efficiency (ANRE)	6.916ns	8.361***	15.139***
Physiological N use efficiency (PNUE)	2.202ns	1.492ns	0.354ns

ns means non-significant effects

*, ** and *** represent significant effects at the P <0.05, P <0.01 and P <0.001, respectively.

### Crop yield and yield components

**Fig 2:**
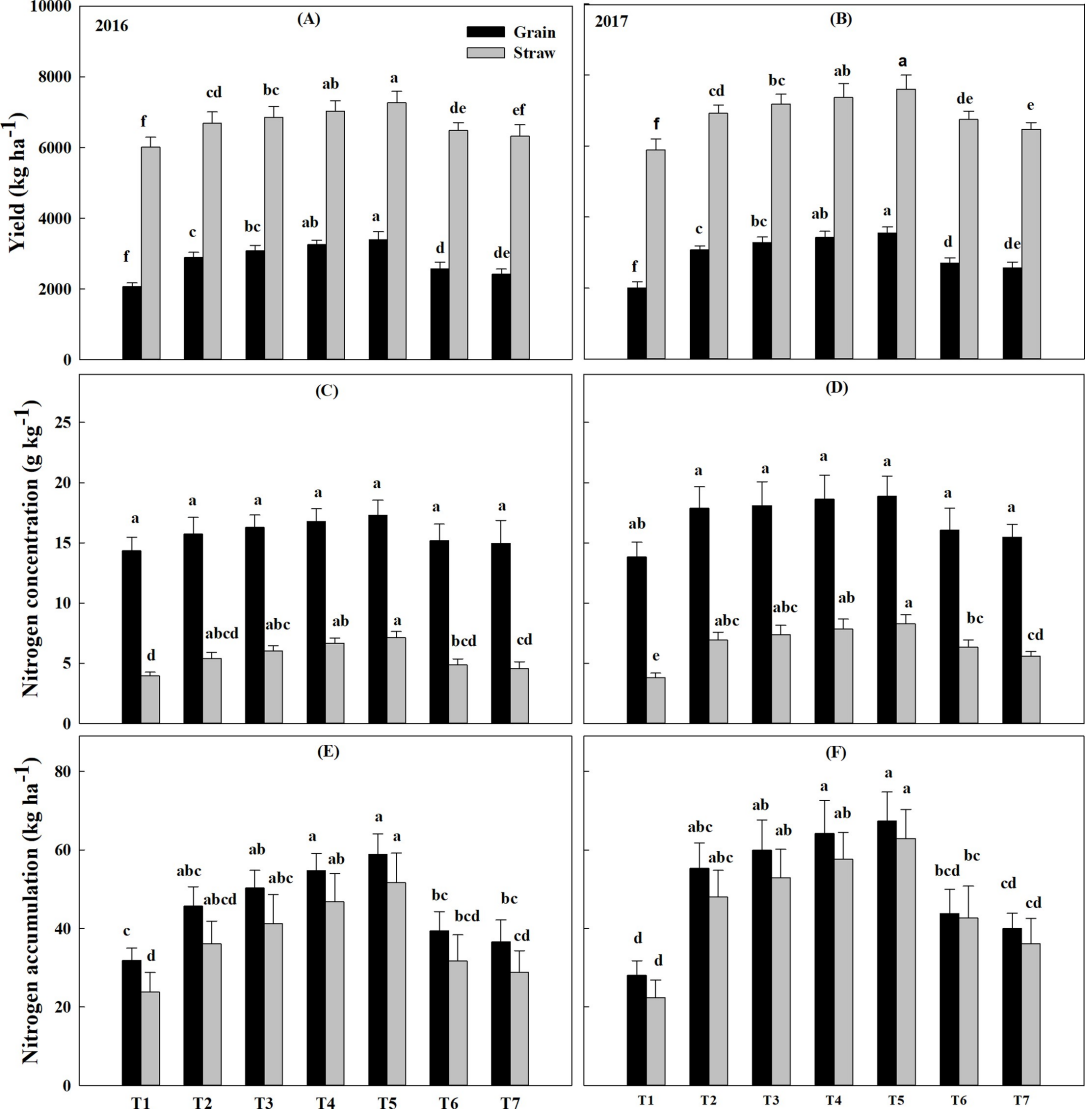
Above ground biomass yield with respect to nitrogen concentration and accumulation under different treatment across the year. (A, B) Changes in above ground biomass yield partitioning in grain and straw during 2016 & 2017, (C, D) changes in partitioning nitrogen concentration in grain and straw during 2016 & 2017, (E, F) changes in partitioning nitrogen accumulation in grain, and straw during 2016 & 2017. Bars indicate standard errors (n = 4). Treatments with different letters for the same variable analyzed (straw or grain) are significantly different according to the LSD test (P <0.05). T1 = Control, T2 = 100% CN, T3 = 100% CN + MBF, T4 = 75% CN + 25% ON + MBF, T5 = 50% CN + 50% ON+ MBF, T6 = 100% ON + MBF and T7 = 100% ON.

We found significantly increased grain yield by the combined application of CN and ON supplemented with MBF which was consistent with the increased number of effective spike per hectare, number of grain per spike and 1000 grain weight under T4 and T5 treatment (**Table 2**). Besides, plant height and ear length were also enhanced significantly under T3, T4 and T5 treatment combinations contributing to higher biomass yield. Grain yield in treatments T2, T3, T4 and T5 was 39%, 48%, 57%, and 64% higher in 2016 whereas 53%, 63%,70% and 77% higher in 2017, respectively as compared to control treatment (T1) (**Fig 2A and 2B**). T5 treatment produced 4% more grain yield than T4 in 2016 and this amount was not less than 3.5% in 2017. Moreover, 17% and 14% more grain yield was produced by T5 treatment in 2016 and 2017, respectively, compared to T2 treatment. The effective spike, grain number per spike and 1000 grain weight were non-significant in T5 treatment with respect to T4 treatment but were significantly higher than either of T2 treatment or T7 treatment across both years (**Table 2**). In both years, straw yield ranged from 6008 to 7263kg ha^-1^ and 5883 to 7599 kg ha^-1^, respectively with T5 being the highest. Furthermore, the replacement of 25% CN with 25% ON in T4 treatment revealed 16.8 and 25% increased straw yield relative to control in 2016 and 2017, respectively (**Fig 2A and 2B**). The replacement of 50% CN with 50% ON reinforced with MBF (T5) showed 20.90% and 29.17% more straw yield than the control in 2016 and 2017, respectively and 4.63% more straw yield in 2017. Above ground biomass yield in T5 was 10658 kg ha^-1^ (in 2016) and 11151 kg ha^-1^ (in 2017) which was approximately 31.98% and 41.33% higher than control (T1) in 2016 and 2017, respectively (**Table 2**). 11.50% more above-ground biomass yield was obtained in T5 treatment than T2 (100% CN) treatment across both years. The harvest index increased from 0.256 (T1) to 0.319 (T5) in the year 2016 while 0.254 (T1) to 0.318 (T5) in the year 2017 with T5 being the highest across both years (**Table 2**).

**Table 2:** Yield and Yield components of oat under different fertilizer treatments.

	Plant height(cm)		Ear length(cm)		Effective Spike Number (10^4^ ha^-1^)		Grain number per spike		1000 grain		Above ground biomass Yield (kg ha^-1^)		Harvest Index	
Treatments									weight (g)					
	2016	2017	2016	2017	2016	2017	2016	2017	2016	2017	2016	2017	2016	2017
T1	81.6b	79.85c	16.4b	15.075d	384h	369d	36.15d	33.4d	23.2d	21.5e	8075f	7890 h	0.256c	0.254d
T2	99.1a	101.85a	19.8ab	23.2ab	483cd	520b	54.6b	59.6b	27.9bc	28.65bc	9572c	9998d	0.302a	0.308 ab
T3	99.75a	103.1a	20.1ab	24.35ab	492bc	535ab	56.75ab	63.2ab	28.6abc	29.85bc	9926bc	10462c	0.309a	0.314a
T4	101.4a	104a	23.1a	25.275a	523ab	590a	59.7ab	68a	30.3ab	32ab	10270ab	10783b	0.317a	0.317a
T5	103.1a	106.1a	21.05ab	26.1a	551a	587a	63.55a	70a	32a	33.95a	10658 a	11151a	0.319a	0.318a
T6	89.4b	94.4ab	18.6ab	20.75bc	452de	473bc	46.75c	51c	26.5bcd	28bc	9049d	9452e	0.284b	0.286bc
T7	86.75b	89.25bc	18ab	18.625cd	431ef	449c	42.8cd	47.85c	25.5cd	27.5cd	8736e	9039f	0.277b	0.284c

In each column, lower case lettering is done to show the significant differences between treatments at P <0.05 level. Values are mean of four replicates. T1 = Control, T2 = 100% CN, T3 = 100% CN + MBF, T4 = 75% CN + 25% ON + MBF, T5 = 50% CN + 50% ON+ MBF, T6 = 100% ON + MBF, T7 = 100% ON

### Nitrogen concentration and accumulation in oat tissues

Our results showed that the reduction of CN and addition of ON supplemented with MBF increased nitrogen concentration in above ground plant parts. Highest N concentrations in grain and straw were recorded in T5 treatment, which were 17.29 g kg^-1^ and 7.12 g kg^-1^ in 2016 while 18.86 g kg^-1^ and 8.28 g kg^-1^ in 2017, respectively. Grain nitrogen concentration was significantly higher than Straw nitrogen concentration across the treatments in both years with control having the least of them (**Fig 2C and 2D**). Consistently, nitrogen accumulation in grain remained significant to nitrogen accumulation to straw in 2016 as well as in 2017 (**Fig 2E and 2F**). The total nitrogen accumulation ranged from 55.62 to 110.59 kg ha^-1^ in 2016 and 50.44 to 130.27 kg ha^-1^ in 2017, respectively. Nitrogen uptake in the treatment T5 was higher than T2 (34.39% in 2016 and 26.08% in 2017) and T6 (54.92% in 2016 and 50.27% in 2017). Above ground N accumulation in T5 was almost double than control in 2016 and even more in 2017.

In case of nitrogen partitioning, the accumulation of N in grain was higher than straw, which ranged from 31.86 to 58.91 kg ha^-1^ in 2016 and 28.03 to 67.35 kg ha^-1^ in 2017.In 2016 N uptake in grain at T5 treatment was 28.82%, 60.95% and 84.90% higher than T2, T7 and control (T1), respectively. On the other hand, grain N accumulation in T5 treatment was 21.68% and 68.41% higher than in T2 and T7 treatment, respectively, and was more than double than control in 2017. N accumulation, in straw showed significant differences among the treatments in both years, ranged from 23.76 to 51.68 kg ha^-1^ and 22.41 to 62.92 kg ha^-1^ in 2016 and 2017, respectively. In 2017 straw N accumulation showed the highest value in T5 treatment which was 21.74% higher than the same treatment in the previous year. Straw N accumulation in control was 6.02% decreased in 2017 compared to 2016 (**Fig 2E and 2F**).

### Nitrogen use efficiency

The components of nitrogen use efficiency (NUE) such as PFP, ANUE and ANRE of oat are shown in **Table 3**. PFP in treatment combination T4 and T5 was higher than the rest of the treatments in our experiment. Although both treatments were non-significant in 2016, but PFP of T5 treatment was significantly higher than T4 in 2017. As far as “ANUE”, “ANRE” and “PNUE” are concerned they remained non-significant between T4 and T5 treatment but significantly higher compared to all other treatment combinations in respective years. Treatment T5 showed highest PFP, ANUE and ANRE ratio, compared with T4, T3, and T2 treatments, indicating gentle decline of NUE with the increasing application of CN. ANRE illustrated highest values (0.55–0.80) in T5 whereas gradual drop in T2 and T3 which were comparable in magnitude with the treatment T4 and T5. Treatment T6 and T7 showed lower PFP, ANUE and ANRE but the higher PNUE than the other treatments. The decrement of CN, increased PNUE in T6 treatment.

**Table 3:** Partial Factor Productivity (PFP), Agronomic Nitrogen use efficiency (ANUE), Apparent N recovery efficiency (ANRE), and physiological N use efficiency (PNUE) by Oat under different fertilizer treatments in 2016 and 2017.

Treatment	PFP (kg kg^-1^)		ANUE (kg kg^-1^)		ANRE (%)		PNUE (kg kg^-1^)	
	2016	2017	2016	2017	2016	2017	2016	2017
T2	95.72 ± 1.32c	99.98 ± 0.98d	14.97 ± 2.16d	21.08 ± 1.80c	26.23 ±4.67cd	52.89 ±5.21bc	59.16 ± 7.81a	40.13 ±1.54a
T3	99.27 ± 1.44bc	104.63 ±0.65c	18.51 ± 1.69bc	25.73 ± 1.21b	35.91 ±5.50bc	62.42 ±4.94ab	53.82 ± 6.72a	41.76 ±3.00a
T4	102.71 ± 1.22ab	107.84 ± 0.88b	21.96 ± 1.61ab	28.94 ± 1.74ab	45.88 ±6.05ab	71.45 ±12.82ab	48.80 ± 2.46a	42.63 ± 4.02a
T5	106.58 ±1.54a	111.51 ± 1.18a	25.83 ± 1.43a	32.61 ± 1.38a	54.97 ±6.54a	79.83 ±11.32a	48.27 ± 3.77a	42.82 ± 4.76a
T6	90.49 ± 1.57d	94.53 ± 0.74e	9.74 ± 2.42de	15.63 ± 0.86d	15.46 ±5.12de	36.07 ±5.73cd	70.44 ± 18.29a	45.62 ± 5.12a
T7	87.37 ± 1.92d	90.40 ± 0.64f	6.61 ± 1.02 e	11.50 ± 0.70e	9.82 ±1.92e	25.68 ±2.28d	59.41 ± 14.13a	45.68 ± 4.22a

In each column, lower case lettering is used to show the significant differences between different types of treatments at P <0.05 level. Values show Standard errors (SE) ± mean of four replicates. T2 = 100% CN, T3 = 100% CN + MBF, T4 = 75% CN + 25% ON + MBF, T5 = 50% CN + 50% ON+ MBF, T6 = 100% ON + MBF, T7 = 100% ON. Standard errors (±) of the means are added with the values & n = 4.

### Nitrogen dynamics in soil

**Fig 3:**
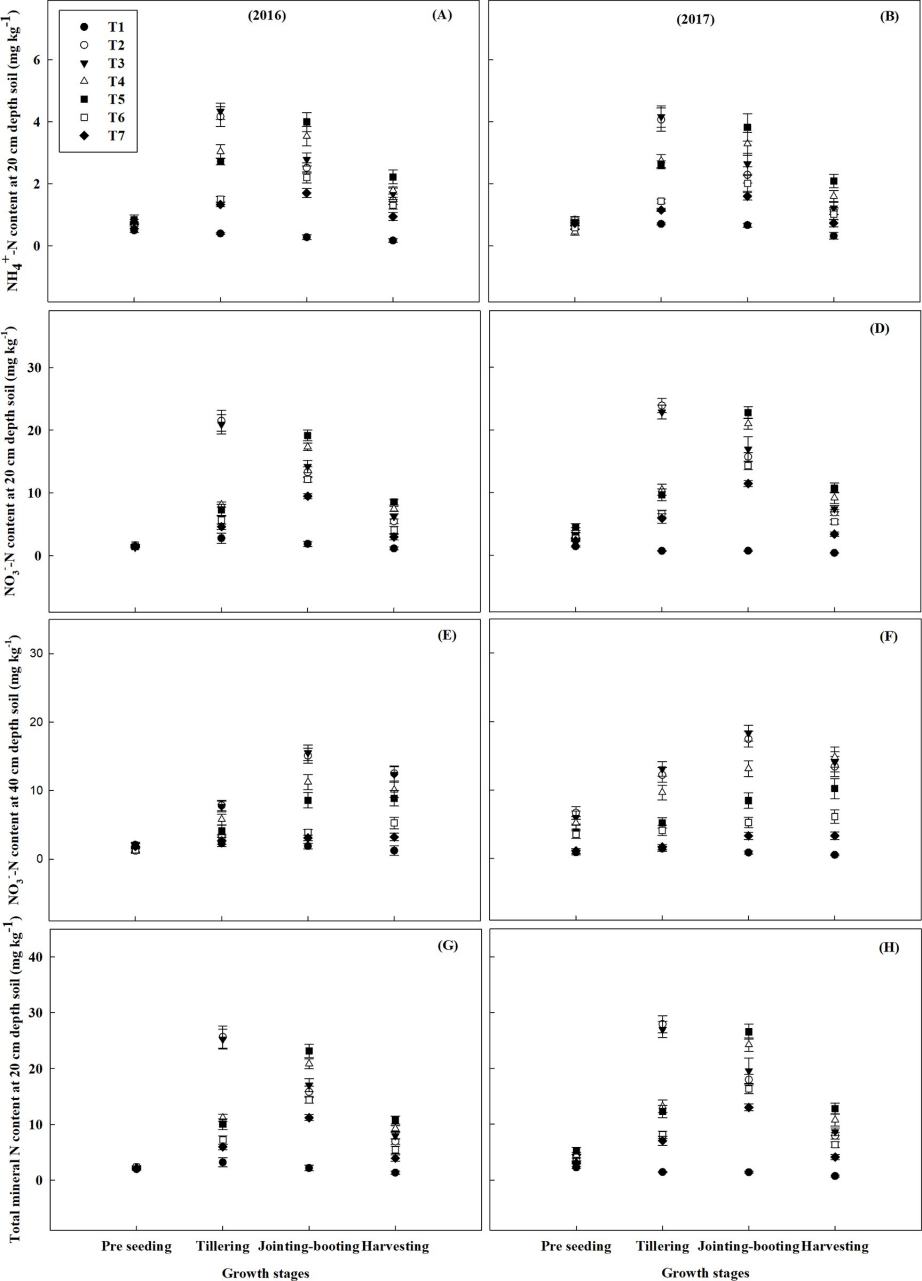
Variations in N content at different depths in different growth stages under different N treatments during 2016 and 2017. (A, B) Ammonium nitrogen (NH_4_^+^-N) content at 20 cm depth soil, (C, D) Nitrate nitrogen (NO_3_^-^-N) content at 20 cm depth soil, (E, F) Nitrate nitrogen (NO3--N) content at 40 cm depth soil and (G, H) Mineral nitrogen (NH_4_^+^-N + NO_3_^-^-N) content at 20 cm depth soil. The bars denote standard deviation of four replicates. T1 = Control, T2 = 100% CN, T3 = 100% CN + MBF, T4 = 75% CN + 25% ON + MBF, T5 = 50% CN + 50% ON+ MBF, T6 = 100% ON + MBF and T7 = 100% ON.

In our experiment, we determined ammonium nitrogen (NH_4_^+^-N) and nitrate nitrogen (NO_3_^-^-N) content of top 20 cm and 40 cm depth soil at four growth stages of the oat. At tillering stage, NH_4_^+^–N content in soil reached the peak point with T3 treatment being the maximum (4.33 mg kg^-1^), then T2 (4.16 mg kg^-1^) and T4 (3.04 mg kg^-1^) treatment in 2016 **(Fig 3A and 3B).** In 2016, the soil NH_4_^+^-N contents in T4 and T5 at jointing- booting stage were 3.53 mg kg^-1^ and 3.99 mg kg^-1^, respectively. NH_4_^+^-N content was the highest in T5 treatment soil at jointing-booting stage and harvesting stage. In both years the trend of NH_4_^+^-N content remained the same except residual NH_4_^+^-N being higher in 2017 than 2016 (**Fig 3A and 3B and S2 Table**). The NO_3_^-^-N contents of top 20 cm soil increased rapidly and were 21.48 mg kg^-1^ in 2016 and 23.86 mg kg^-1^ in 2017 for T2 treatment, respectively. Soils of T2 and T3 treatment observed decrement of NO_3_^-^-N contents at jointing-booting stage which were increased rapidly in T4 and T5 treatment at the same depth. Generally, NO_3_^-^-N content of top 20 cm soil increased in all the treatments except T1, T2 and T3 till jointing-booting stage but decreased at the harvesting stage. At jointing-booting stage, NO_3_^-^-N content was the highest (19.16 and 22.76 mg kg^-1^, in both years, respectively) in soil of T5 treatment at 20 cm soil depth (**Fig 3C and 3D**). On the other hand, NO_3_^-^-N content at 40 cm soil depth was significantly higher in T2 and T3 treatment at every stage, maximizing at jointing-booting stage **(Fig 3E and 3F).** However, the total mineral N (NH_4_^+^-N + NO_3_^-^-N) at 20 cm soil depth was highest at T2 and T3 treatment (25.64 and 25.25 mg kg^-1^ in 2016, and 27.92 and 26.99 mg kg^-1^ in 2017, respectively) at tillering stage, but at jointing-booting stage it was the highest in T5 treatment (**Fig 3G and 3H**).

### Soil pH, Total N, Organic C, Dissolved Organic Nitrogen (DON) and Dissolved Organic Carbon (DOC)

The diverse nitrogen sources illustrated a significant effect on fertility status of the experimental soil without affecting soil pH too much. Sole application of CN did not improve pH and fertility status of the differentially treated soils rather, it slightly decreased the soil pH. Combined use of CN and ON with MBF neutralized the acidifying effect of CN and revealed an increasing tendency of soil pH (**Table 4**). Total Nitrogen (TN) and organic carbon (OC) content of the soil during 2017 harvesting time were analyzed (**Table 4 and S2 Table)**. The levels of TN and OC content varied markedly among the different treatment soil after harvesting of oat in 2017. The highest level of soil TN was observed in T2 and T3, followed by T4 treatment, without significance. Contrarily, compared to control, 77.92% and 66.23% more TN was observed in T2 and T5 treatments, respectively. On the other hand, organic C contents in soil were the highest in the treatments T6 and T7followed by T5 treatment. The SOC lowered with the increasing CN and vice versa whereas dissolved organic nitrogen (DON) and dissolved organic carbon (DOC) differed significantly between treatments in both years. DON and DOC in T6 treatment were higher by 63.41% and 35.1%, respectively, compared to T3 treatment (**Table 4**).

**Table 4:** Soil chemical properties after 2^nd^ harvesting of Oat. Bulk pH, soil total nitrogen and soil organic carbon, dissolved organic nitrogen (DON) and dissolved organic carbon (DOC) under different fertilizer treatments.

Treatments	Soil pH (1:2.5 H_2_O)	Total Soil N (g kg^-1^ soil)	SOC (g kg^-1^)	DON (mg kg^-1^)	DOC (mg kg^-1^)
T1	7.4±0.14a	0.77 ±0.08cd	6.31±0.64f	17 ± 2.19d	59 ± 3.9e
T2	6.9±0.07a	1.37 ±0.08ab	9.21±0.63e	27 ±2.9d	68 ± 3.3d
T3	6.8±0.08a	1.43 ±0.05a	13.03±0.87d	41 ± 3.1c	97 ± 4.1c
T4	7.1±0.15a	1.34 ±0.08ab	17.27±0.68c	44 ± 2.7c	108 ± 4.9b
T5	7.5±0.14a	1.28±0.05ab	18.26±0.77bc	61 ± 4.2a	119 ± 5.4a
T6	7.7± 0.18a	1.22 ±0.05ab	22.30±0.95a	67 ± 4.9a	131 ± 7.1a
T7	7.4±0.07a	1.15 ±0.06bc	20.01±0.81de	52 ± 3.7b	127 ± 5.2a

In each column lower case lettering is used to show the significant differences between different types of treatments at P <0.05 level. Values show Standard errors (SE) ± mean of four replicates. T1 = Control, T2 = 100% CN, T3 = 100% CN + MBF, T4 = 75% CN + 25% ON + MBF, T5 = 50% CN + 50% ON+ MBF, T6 = 100% ON + MBF, T7 = 100% ON

### Soil microbial biomass and soil respiration

**Fig 4:**
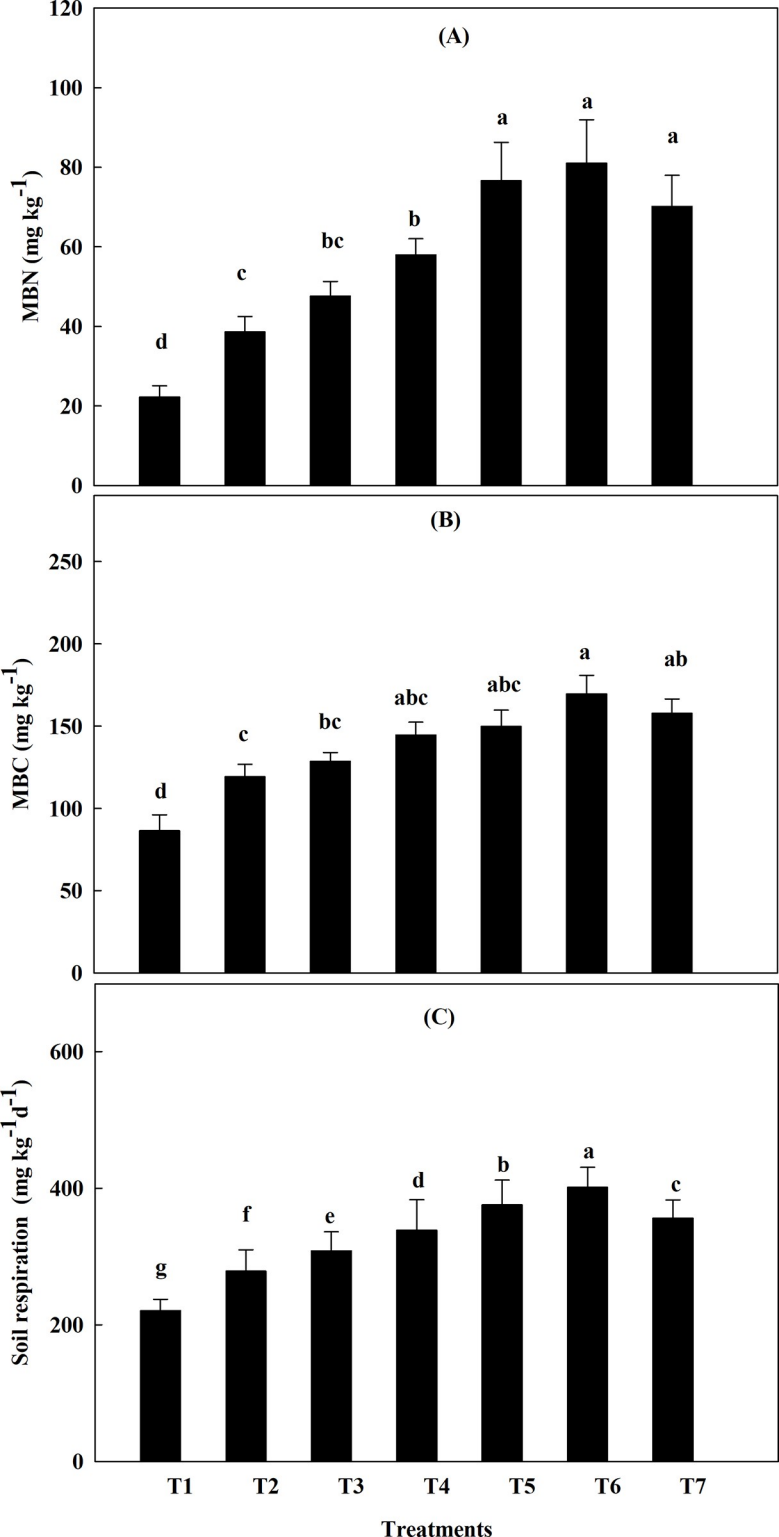
Soil Microbial biomass and respiration rate. Mean values (2016–17) of (A) Soil microbial biomass N (MBN), (B) soil microbial biomass C (MBC) and (C) soil respiration rate, in different treatment during the jointing booting stage of oat. Bars indicate standard errors (n = 4). Treatments with different letters are significantly different according to the LSD test (P <0.05). T1 = Control, T2 = 100% CN, T3 = 100% CN + MBF, T4 = 75% CN + 25% ON + MBF, T5 = 50% CN + 50% ON+ MBF, T6 = 100% ON + MBF and T7 = 100% ON.

Soil microbial biomass nitrogen (MBN) and microbial biomass carbon (MBC) under different treatment at jointing-booting stage were represented as mean values of two years in **Fig 4A and 4B.** MBC increased significantly in the treatment fertilized with ON+MBF and only ON against their control during the jointing-booting stage, with the highest increment of MBC consistently occurring in the T6 treatment **(Fig 4B).** Difference between T6 and other treatments for MBC was highly significant except T7. The different N sources also affected MBN in soil, which varied between different treatments (**Fig 4A**). Similarly, treatment T6 had the highest MBN content compared to other treatments. Nevertheless, no significant differences were displayed by T5, T6 and T7 treatments. Soil with different fertilization treatments showed diverse respiration rates and higher cumulative CO_2_ production compared to those found in control treatment (**Fig 4C**). A significantly higher respiration rate was observed in T6 treatment, whereas T5 and T7 were at sequentially lower order compared to other treatments. The respiration rate in T6 was approximately 82% higher than in control. Similarly, compared to treatment T2 28% more respiration rate was observed in T7 treatment.

### Enzyme activity

In current study, we analyzed dehydrogenase, acid phosphatase, arylsulphatase, ß-glucosidase and BAA-protease in soil under applied treatments. Soil under T5, T6 and T7 treatments showed high levels of aforementioned enzyme activities with dehydrogenase showing significantly higher activity at T6 treatment than any other treatment except T7 **(Table 5)**. Acid phosphatase activity was significant at T5 treatment with T6 being non-significant to T5. Arylsulphatase, ß-glucosidase and BAA-protease activities were higher in T5, T6 and T7 treatments compared to all other treatments but the effect remained non-significant within themselves (**Table 5**).

**Table 5:** The enzymatic activities in the soil under different treatments during the jointing-booting stage in 2017.

Enzyme	T1	T2	T3	T4	T5	T6	T7
Dehydrogenase (nmol TPFg^-1^ soil h^-1^)	140±4.98f	157±5.91ef	187±9.9d	219±8.1c	254±11.43ab	269±8.45a	231±6.26bc
Acid phosphatase (μmol p-nitrophenol g^-1^ h^-1^)	6.1±0.42g	8.2±0.93f	11.2±0.99de	14.8±1.2bc	18.7±1.61a	16.2± 1.43ab	13±1.8cd
Arylsulphatase (μmol p-nitrophenol g^-1^ h^-1^)	0.24±0.007c	0.28±0.002c	0.37±0.007bc	0.41±0.01abc	0.59± 0.02a	0.61±0.01a	0.49±0.009ab
ß-glucosidase (μmol p-nitrophenol g^-1^ h^-1^)	2.5±0.04c	2.9±0.06c	3.61±0.1abc	4.3±0.07abc	5.4±0.09a	5.1±0.1ab	4±0.21abc
BAA-protease (μmol NH_3_- N g^-1^ h^-1^)	4.9±0.41c	5.43±0.6bc	5.71± 0.45bc	6.51± 0.34abc	7.8±0.83a	7.97±0.79a	6.91±0.71ab

In each column lower case lettering is used to show the significant differences between different types of treatments at P <0.05 level. Values show Standard errors (SE) ± mean of four replicates. T1 = Control, T2 = 100% CN, T3 = 100% CN + MBF, T4 = 75% CN + 25% ON + MBF, T5 = 50% CN + 50% ON+ MBF, T6 = 100% ON + MBF, T7 = 100% ON

## Discussions

### Yield attributes influenced by different amendments

The average grain and straw yield with nitrogen use efficiency over two years indicated that different treatments of nitrogen applied either alone or combined in different proportions, showed significant differences in grain and biomass yield, and in the nitrogen recovery rate. In this study higher oat grain and straw yield were obtained by the combined use of CN and ON with MBF as evident from increased number of effective spikes per hectare, number of grains per spike and 1000 grain weight **(Table 2)** and this finding is consistent with the previous work result [44]. On the whole, yield results indicated that application of CN and ON with MBF could significantly increase oat yield compared to 100% CN alone. Higher grain yield and 1000 grain weight in the 100% CN + MBF (T3) treatment as compared to 100% CN (T2) whereas lower grain yield in 100% ON compared to 100% ON+ MBF in 2016 and 2017 respectively **(Fig 2A and 2B)** were consistent with previous experiment which reported that maize grain yield and 1000-grain weight were increased 13% and 10% respectively, with the application of MBF + 88kg N ha^-1^ in comparison with the 100% dose (175 kg N ha^-1^) of CN [45]. Moreover, the yield results of current study illustrated the effectiveness of MBF when applied with CN and ON owing to the fact that the application of microbial fertilizer could significantly increase the number of tillers per plant (10–21%), straw yield (22–39%) and finally grain yield (15–43%) [46] as compared to individual application of MBF or ON [47,48,49,50]. It has also been reported that the application of MBF along with organic and chemical fertilizers increased the growth of cotton and maize [51,52]. Consistent with several previous reports, our findings further elaborated that higher yield cannot be achieved solely depending on organic manure [49,53]. In this experiment higher grain and straw yield under combined application of CN and ON with MBF would have resulted from enough nutrient removal and improved retention capacity of soil [49,54]. Furthermore, organic manure releases N slowly into the soil which could really match with the demand of N in different growth stages of crop, and this is very important off late to ameliorate environmental problems by reducing the application of CN and establishing organic agriculture [48,50,55]. Previously, researchers used 50% less mineral NPK with OM and effective MBF in cotton field and saved 50% mineral N compared to the use of full dose of NPK [52]. The higher grain and biomass yield due to the application of MBF supplemented with organic and chemical fertilizer could also be explained by the possible involvement of N_2_-fixation mechanism [56,57], excretion of ammonia [58], solubilization of phosphorous [59] and production of growth hormones [60].

### Plant mineral contents and Nitrogen Use Efficiency (NUE)

Plants uptake N either in the form of nitrate or ammonium depending upon their availability in the soil solution. Many reasons can influence their availability, one of them is soil organic matter and the second one is microbial activity [27]. It is essential to coordinate crop N demand and supply to improve the NUE. In our study, NUE was higher as is obvious from increased PFP, ANUE and ANRE in T5 treatment compared to all other treatments across the years (**Table 3**). 50% CN + 50% ON + MBF (T5) treatment improved the nitrogen recovery and uptake compared to full dose of CN (T2) (**Table 3**) consistent with rice and wheat [54,55,61], elaborating a positive interaction between organic and mineral N reinforced by MBF to reduce nitrogen loss in the environment and increase its efficiency [27]. On the other hand, the use of MBF could have increased the nutrient accumulation in oat similar to various crops such as maize [62], cotton, pea [63], and wheat [64]. Nitrogen accumulation in plant is associated with higher biomass production including greater N concentration in plant. The increased N accumulation in plants due to the combined application of chemical and organic fertilizer with MBF might be interpreted by N fixation mechanisms, solubilization of phosphorous, production of IAA of bacterial strains [65]. Approximately 8–10% and 9–18% increase in grain and straw nitrogen in both years, respectively (**Fig 2E and 2F**) showed the effectiveness of microbes to make nitrogen available to crop plants for uptake [66]. Higher N efficiency was found in T5 treatment indicating that the level, sources and timing of applied nitrogen firmly matched the N demand of the oat crop. This N level reduced the loss of nitrogen in the environment through leaching, runoff or other possibly associated mechanism during critical period of oat growth (jointing-booting stage) and increased the uptake of N and hence, oat yield. Additionally, as CN was applied with ON and MBF in the combination treatment, initial N requirement of the oat was ensured by CN whereas, slow mineralization of OM and likely increased N fixation as indicated by higher abundance of *nif*H gene **(S3 Fig)** with higher enzymatic activities **(Table 5)**, supplied N during the later growth stages of oat. This regulation of nitrogen supplies under T5 treatment favored the mineral composition of oat for effective biomass and yield production (**S4 Table**).

### Fate of nitrogen applied in soil

To achieve sustainability in agriculture, it is now mandatory to reduce N loss and manage high yielding crops properly [67]. In previous studies, it was observed that the losses of N were higher with the increased doses of applied CN up to 200 kg ha^-1^ [68]. Ammonia volatilization is the most crucial way of N loss during the growing season [67,69], while leaching and denitrification are the other ways of N loss. The application of ON is an effective way to mitigate NH_3_ volatilization losses [55,69,70]. As illustrated by our experiment where NH_4_^+^-N content of the top 20 cm soil increased in CN+ ON+ MBF (T5) and ON+MBF (T6) treatment combinations which might be due to the mineralization of OM resulted from increased microbial activity by MBF. Nitrogen mineralization from poultry manure was the highest at jointing-booting stage of the crop (50–65 Days after sowing) **(Fig 3A and 3B)** which is consistent with the previous studies where highest mineralization of N was observed after 63 days of incubation involving poultry manure [71]. Nitrate (NO_3_^-^) is often leached down from the rhizosphere and deposited below at 40 cm depth as is obvious from the low and high NO_3_^-^- N contents at 20cm and 40cm soil depth in treatments T2 and T3 during the jointing booting stage in our experiment **(Fig 3C–3F)**. The highest NO_3_^-^-N content and total mineral N at 20cm depth during critical jointing-booting stage of oat across the years **(Fig 3C–3D and 3G–3H)** further emphasized the point that combined use of ON and CN reduce the N loss by transforming inorganic N to organic N forms (immobilization), thereby increase the fertilizer efficiency compared with the sole application of CN [72].

### Microbial biomass C and N

Microbial biomass is a crucial component of organic matter ensuring plant nutrient bioavailability [73]. Distinguishable differences in soil microbial biomass level were found between the treatments in jointing-booting stage, with highest level in the treatment T6; and then T5 and T7 were in the sequential lower order (**Fig 4A and 4B**). The C pool in the organic manure, which can be easily metabolized, contributed to the soil microbial biomass [74,75], thereby affecting crop growth & development directly or indirectly [76]. Soil microbial activity enhancement was closely linked with high availably of soil N for crop plants [77]. Substantial increments in MBN and MBC in treatments T6 whereas decrement in treatment T2 elicited the impact of combined application of OM and MBF (**Fig 4A and 4B**). We speculated that it might be due to the balance between organic and inorganic pools of nitrogen in the soil, which acted as a substrate for MBF to mineralization and immobilization available N reserves to ensure a favorable C/N ratio upon which oat could dwell. Microbial activity is directly related to soil respiration [78] which in our experiment was highest in T6 treatment **(Fig 4C)** at jointing-booting stage. Respiration is considered to reflect the availability of C for microbial maintenance and is a measure of the basic turnover rates in soil [79]. We reasoned that amended soil with PM and MBF derived substantial microbial biomass effective for mineralization and thus increased soil available N for the oat.

### Dehydrogenase and other soil hydrolytic enzyme activity

Microbial enzymes in the soil are actually induced by metabolic procedure, generally reflecting the soil microbial activity level and the biochemical reaction intensities [22]. Current study illustrated that different fertilizer treatments undoubtedly affected the enzyme activities in soil (**Table 5**). Dehydrogenase (DHA) is an important indicator of microbial activity among the oxyreductase enzyme, and has been treated as a biomarker to assess quality of soil under cultivation [80]. In this experiment, compared to T2 treatment more than 60% and 70% higher DHA activity was observed in T5 and T6 treatment respectively, which is highly significant. Similar trend was observed for other enzymes with increased activities in T5 and T6 treatments **(Table 5),** mainly due to enhanced microbial activity when combined fertilization was practiced [81]. Greater DHA activity is the indicator of an ample availably of organic degradable substrates in the soil, resulting in higher microbial activities. Dehydrogenase enzyme had a positive correlation with SOC (P<0. 01, n = 21 and r = 0.95) and soil respiration (P<0. 01, n = 21 and r = 0.977) (Data not shown) which means increased microbial growth and activity due to organic substrate in the soil [82,83]. Enhanced activities of hydrolytic enzymes such as acid phosphatase, arylsulphatase, ß-glucosidase also indicated that soil function was enhanced with the incorporation of OM and MBF. On the whole, our results indicated that metabolic processes were higher in the OM and MBF treated soil as compared to control or soil treated with CN only, which affected biological transformation of soil N. Apart from reducing nitrogen losses and increasing nitrogen use efficiency in oat production system, the findings of current study can be combined with various environmental factors to calibrate or even generate resource use efficient models for oat as well as other cereal crops in future [84,85,86].

## Conclusion

It is necessary to understand the economics and environmental hazards associated with N losses in agricultural fields. Experimental results of current study revealed the benefits of combined application of CN, ON and MBF in terms of enhanced biomass and grain yield of oat, owing to the betterment of soil properties in semi-arid regions of Northeast China. Integrated application of CN, ON and MBF increased nitrogen availability in the rhizosphere and reduced N loss, perhaps due to slow release of N from ON and on-time availability during the most critical period of oat growth. Moreover, the increased microbial activity due to organic N and MBF improved soil health in terms of soil respiration and contributed to microbial N pool available to oat for uptake and translocation. Overall, we found 50% CN+50% ON+MBF as an effective fertilizer dose to minimize nitrogen loss, and maximize NUE and oat production in semi-arid region of China.

## Supporting information

S1 Fig The daily average temperature and precipitation during the oat growing period in 2016 and 2017. (TIF)

S2 Fig **Microbial community structure of microbial fertilizers (A) phylum and (B)genus level**.
(TIF)

S3 Fig **Abundance of *nif*H gene in rhizosphere soil samples under different treatments (A), taxonomic distribution of the diazotrophic Phyla in different rhizosphere soil samples (B) and percentage of α, β and γ Proteobacteria in different rhizosphere soil samples (C)**. Values are mean ± SD (n = 3). Different letters indicate significant differences (P<0.05). T1 = Control, T2 = 100% CN, T3 = 100% CN + MBF, T4 = 75% CN + 25% ON + MBF, T5 = 50% CN + 50% ON+ MBF, T6 = 100% ON + MBF and T7 = 100% ON at the 2nd year jointing booting stage of oat, respectively.
(TIF)

S1 Table The physical and chemical properties of the soil in experimental field. (DOCX)

S2 Table Soil chemical properties after 2nd harvesting of Oat. Soil pH, Soil total nitrogen, NH_4_^+^-N and NO_3_^-^ -N at different soil depths under different fertilizer treatments.
(DOCX)

S3 Table Fertilizer balance under different treatments. (DOCX)

S4 Table Minerals content in Shoot and root after 2^nd^ year harvesting of oat under different treatment. (DOCX)
