# Supplementary figures and images for: Enhancing naked oat (Avena nuda L.) productivity with minimal indirect nitrogen loss and maximum nitrogen use efficiency through integrated use of different nitrogen sources
Source: PLoS One. 2019 Mar 18;14(3):e0213808. doi: 10.1371/journal.pone.0213808 (PMC6422306; doi:10.1371/journal.pone.0213808)

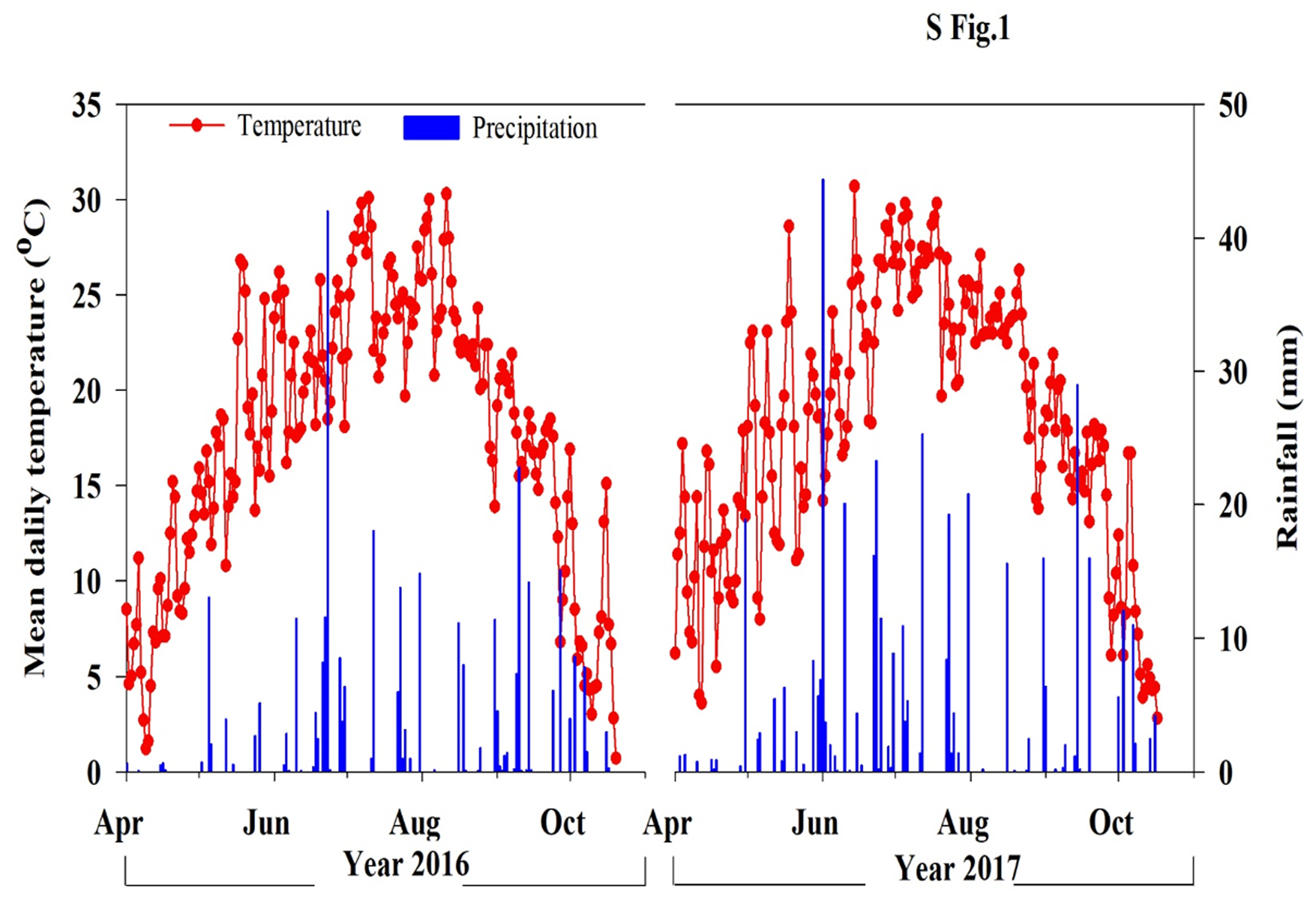

Supplement: S1 Fig — (TIF) [file pone.0213808.s001.tif]

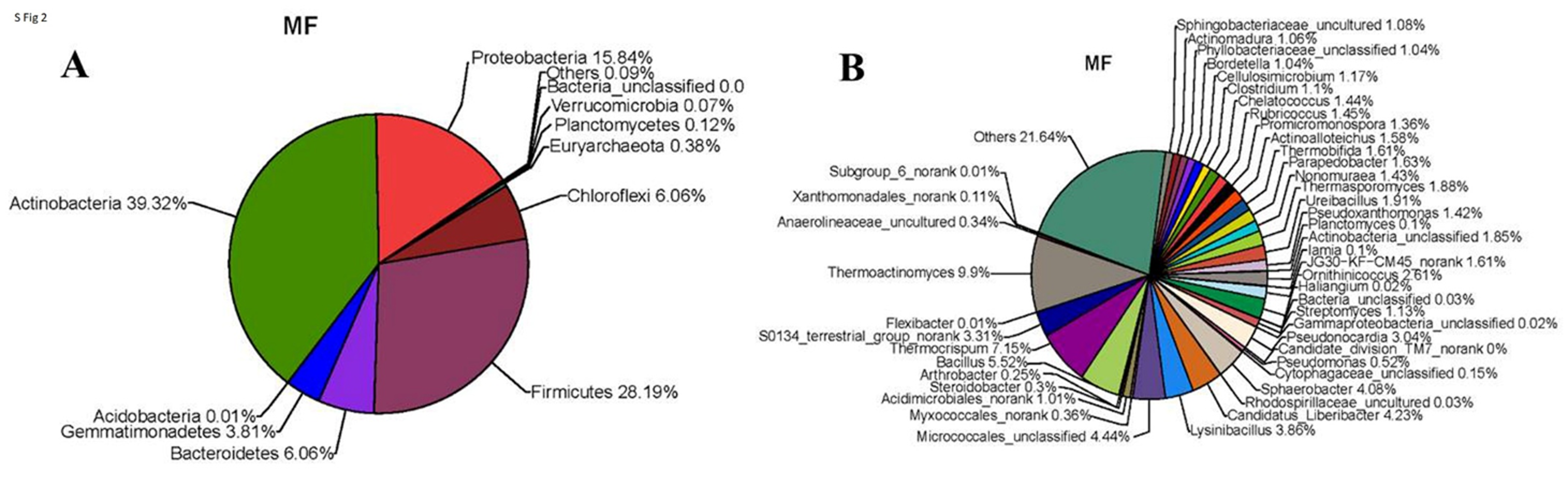

Supplement: S2 Fig — Microbial community structure of microbial fertilizers (A) phylum and (B)genus level. (TIF) [file pone.0213808.s002.tif]
