# Supplementary material for: Enhancing naked oat (Avena nuda L.) productivity with minimal indirect nitrogen loss and maximum nitrogen use efficiency through integrated use of different nitrogen sources
Source: PLoS One. 2019 Mar 18;14(3):e0213808. doi: 10.1371/journal.pone.0213808 (PMC6422306; doi:10.1371/journal.pone.0213808)
Supplement: S1 Table — (DOCX) [file pone.0213808.s004.docx]

**S1 Table** **|** The physical and chemical properties of the soil in experimental field.

| Soil Properties | Values | |
| --- | --- | --- |
|  | 2016 | 2017 |
| Bulk density(gm^-3^) | 1.34 ± 0.04 | 1.4 ± 0.03 |
| Sand (g kg^-1^) | 32.81 ± 3.50 | 34.53 ± 4.23 |
| Silt (g kg^-1^) | 39 ± 4.47 | 37.39 ± 3.75 |
| Clay (g kg^-1^) | 28.44 ± 2.25 | 27.69 ± 3.33 |
| Soil pH(1:2.5 H_2_O) | 7.7 ± 0.17 | 7.2 ± 0.20 |
| Organic carbon(g kg^-1^) | 7.59 ± 0.78 | 11.8 ± 0.79 |
| Total N(g kg^-1^) | 0.54 ± 0.06 | 0.74 ± 0.04 |
| NH_4_^+^-N(mg kg^-1^) | 0.66 ± 0.09 | 0.71 ± 0.08 |
| NO_3_^-^-N(mg kg^-1^) | 1.5 ± 0.13 | 2.94 ± 0.27 |
| Available P(mg kg^-1^) | 14.17 ± 1.27 | 17.20 ± 1.78 |
| Available K(mg kg^-1^) | 48.57 ± 4.31 | 53.3 ± 3.99 |
| Cation exchange capacity(CEC) cmol^(+)^ kg^-1^ soil | 12.2 ± 1.54 | 13.33 ± 1.66 |

The textural classification of the soil was according to USDA classification system

Standard errors (±) of the means are added with the values & n=4.
