# Supplementary material for: Enhancing naked oat (Avena nuda L.) productivity with minimal indirect nitrogen loss and maximum nitrogen use efficiency through integrated use of different nitrogen sources
Source: PLoS One. 2019 Mar 18;14(3):e0213808. doi: 10.1371/journal.pone.0213808 (PMC6422306; doi:10.1371/journal.pone.0213808)
Supplement: S2 Table — Soil pH, Soil total nitrogen, NH4+-N and NO3- -N at different soil depths under different fertilizer treatments. (DOCX) [file pone.0213808.s005.docx]

**S2 Table |** Soil chemical properties after 2nd harvesting of Oat. Soil pH, Soil total nitrogen, NH_4_^+^-N and NO_3_^-^ -N at different soil depths under different fertilizer treatments

| Treatment | Soil Total N | | |  |  | NH_4_^+^-N | |  | NO_3_^-^ -N |  | Soil pH | | |
| --- | --- | --- | --- | --- | --- | --- | --- | --- | --- | --- | --- | --- | --- |
|  | 10 cm | 20 cm | 40 cm |  | 10 cm | 20 cm | 40 cm |  | 10cm |  | (1:2.5 H_2_O) | (1:2.5 KCl) | (1:5CaCl_2_) |
| T1 | 0.51 ±0.03d | 0.77 ±0.08cd | 1.22 ±0.03g |  | 0.25 ±0.013f | 0.32 ±0.11f | 0.19 ±0.02d |  | 0.33 ±0.01g |  | 7.4±0.14a | 6.8 ±0.17a | 6.9 ± 0.22a |
| T2 | 0.94 ±0.04a | 1.37 ±0.08ab | 2.54 ±0.03b |  | 1.01 ±0.01d | 1.12 ±0.11c | 0.68 ±0.04a |  | 6.24 ±0.26d |  | 6.9±0.07a | 6.3 ±0.12b | 6.4 ±0.23b |
| T3 | 0.99 ±0.06a | 1.43 ±0.05a | 2.67 ± 0.04a |  | 1.34 ±0.28c | 1.22 ±0.20c | 0.67 ±0.04a |  | 7.051 ±0.10c |  | 6.8±0.08a | 6.4 ±0.13b | 6.3 ±0.22b |
| T4 | 0.88 ±0.06ab | 1.34 ±0.08ab | 2.41 ±0.02c |  | 1.44 ±0.02b | 1.60 ±0.19b | 0.48 ±0.03b |  | 8.36 ±0.29b |  | 7.1±0.15a | 6.8 ±0.23a | 6.9 ±0.12a |
| T5 | 0.79 ±0.03bc | 1.28±0.05ab | 2.29 ±0.04d |  | 1.88 ±0.01a | 2.09 ±0.22a | 0.44 ±0.04bc |  | 9.98 ±0.33a |  | 7.5±0.14a | 6.9 ±0.23a | 7.1 ±0.11a |
| T6 | 0.74 ±0.04bc | 1.22 ±0.05ab | 2.01 ±0.05e |  | 1.00 ±0.01d | 1.02±0.17cd | 0.26 ±0.03d |  | 4.99 ±0.13e |  | 7.7± 0.18a | 7.1 ±0.12a | 7 ±0.12a |
| T7 | 0.71 ±0.04c | 1.15 ±0.06bc | 1.89 ±0.04f |  | 0.69 ±0.02e | 0.73 ±0.11e | 0.39 ±0.02c |  | 3.01 ±0.08f |  | 7.4±0.07a | 7 ± 0.12a | 7.2 ±0.12a |

In each column lower case lettering is used to show the significant differences between different types of treatments at P <0.05 level. Values show Standard errors (SE) ± mean of four replicates. T1= Control, T2= 100% CN, T3= 100% CN + MBF, T4= 75% CN + 25% ON + MBF, T5= 50% CN + 50% ON+ MBF, T6= 100% ON + MBF, T7= 100 % ON.
