# Supplementary material for: Enhancing naked oat (Avena nuda L.) productivity with minimal indirect nitrogen loss and maximum nitrogen use efficiency through integrated use of different nitrogen sources
Source: PLoS One. 2019 Mar 18;14(3):e0213808. doi: 10.1371/journal.pone.0213808 (PMC6422306; doi:10.1371/journal.pone.0213808)
Supplement: S3 Table — (DOCX) [file pone.0213808.s006.docx]

| Treatment | N (Kg ha^-1^) | P (Kg ha^-1^) | K (Kg ha^-1^) | Poultry manure  (Kg ha^-1^) | Microbial Fertilizer (Kg ha^-1^) |
| --- | --- | --- | --- | --- | --- |
| Control | - | 458.33  (P_2_O_5_ =55kg) | 90  (45 K_2_O) |  | - |
| 100% CN | 194.38 | 458.33 | 90 |  | - |
| 100% CN +MBF | 194.38 | 458.33 | 90 |  | 20 |
| 75% CN + 25% ON + MBF | 145.79 | 376.325 | 76.96 | 1204.5  (P=9.84,K= 6.52) | 20 |
| 50% CN + 50% ON + MBF | 97.19 | 294.33 | 63.94 | 2408.99  (P=9.84,K= 6.52) | 20 |
|  |  |  |  |  |  |
| 100% ON + MBF | - | 130.33 | 37.86 | 4817.99  (P=39.36,K= 26.07) | 20 |
| 100% OM | - | 130.33 | 37.86 | 4817.99  (P=39.36,K= 26.07) | 20 |

**S3 Table |** Fertilizer balance under different treatments.
