# Supplementary material for: Enhancing naked oat (Avena nuda L.) productivity with minimal indirect nitrogen loss and maximum nitrogen use efficiency through integrated use of different nitrogen sources
Source: PLoS One. 2019 Mar 18;14(3):e0213808. doi: 10.1371/journal.pone.0213808 (PMC6422306; doi:10.1371/journal.pone.0213808)
Supplement: S4 Table — (DOCX) [file pone.0213808.s007.docx]

|  | **Treatment** | **P** | **K** | **Ca** | **Mg** | **Fe** | **Na** | **Cu** | **Zn** | **Mn** | **S** | **B** |
| --- | --- | --- | --- | --- | --- | --- | --- | --- | --- | --- | --- | --- |
|  |  | **(mg kg^-1^)** | | | | | | | | | | |
| Shoot | T1 | 1571.67d | 7750f | 3120e | 1768.33e | 96c | 1848.33g | 6.17c | 16.97e | 34.02e | 1253.33d | 3.78d |
|  | T2 | 2281.67bc | 9900c | 3690c | 2290c | 91.17c | 6050b | 12.61a | 20.27d | 58.67b | 1410c | 4.71c |
|  | T3 | 2391.67b | 10550b | 3938.33b | 2311.67b | 94.5b | 6666.67a | 14.61a | 21.67c | 65.17a | 1566.67b | 5.5b |
|  | T4 | 2603.33a | 10800b | 4843.33a | 2358.33b | 133.5b | 4741.67c | 12.31a | 22.55c | 54.17cd | 1583.33b | 5.78b |
|  | T5 | 2680a | 12816.67a | 3961.67b | 2495a | 152.5ab | 3348.33d | 11.16ab | 23.8b | 56.5bc | 1610ab | 7.2a |
|  | T6 | 2220bc | 9500d | 3631.67c | 2188.33c | 165a | 3001.67e | 10.71ab | 25.15a | 53.87cd | 1663.33a | 5.43b |
|  | T7 | 2103.33c | 9016.67e | 3450d | 2021.67d | 146.5ab | 2673.33f | 7.51bc | 24.83ab | 52.57d | 1620ab | 4.48c |
| Root | T1 | 572.5g | 5900f | 7325e | 2915e | 3900g | 622.5e | 16.52f | 24.5f | 137.5g | 388e | 27.17d |
|  | T2 | 720d | 7650d | 12400c | 3615d | 5450f | 1040c | 39.74b | 50.5b | 189.5b | 573.5d | 40.60c |
|  | T3 | 950c | 8325c | 13925b | 3707.5cd | 6050e | 1282.5b | 43.49a | 56.75a | 208.5a | 644.75cd | 42.70b |
|  | T4 | 1075b | 9625b | 14350b | 3795bc | 6650d | 1680a | 28.99c | 48.78bc | 174.5c | 697.5c | 44.995a |
|  | T5 | 1105a | 10750a | 15025a | 3905ab | 8200b | 1760a | 22.19d | 46.95c | 168.25d | 875b | 45.22a |
|  | T6 | 687.5e | 7550d | 12225c | 3972.5a | 9100a | 872.5d | 20.42de | 43.4d | 161e | 1075a | 39.50c |
|  | T7 | 645f | 6950e | 11425d | 3855ab | 7400c | 762.5d | 19.97e | 37.75e | 152f | 895b | 38.95c |

**S4 Table |** Minerals content in Shoot and root after 2^nd^ year harvesting of oat under different treatment.

Note: In each column lower case lettering is used to show the significant differences between different types of treatments at P <0.05 level. T1= Control, T2= 100% CN, T3= 100% CN + MBF, T4= 75% CN + 25% ON + MBF, T5= 50% CN + 50% ON+ MBF, T6= 100% ON + MBF, T7= 100 % ON.
